# Supplementary material for: Dynamics of IgM and IgA Antibody Response Profile Against Vibrio cholerae Toxins A, B, and P
Source: Int J Mol Sci. 2025 Apr 9;26(8):3507. doi: 10.3390/ijms26083507 (PMC12027190; doi:10.3390/ijms26083507)
Supplement: Supplementary file 1 [file ijms-26-03507-s001.zip › ijms-3387352-supplementary captions.pdf]

Figure S1; List of synthesized peptides covering the entire sequence of *Vibrio cholera* toxin A (P01555), toxin B (P01556);

Figure S2: ELISA using as antigen the Tx45-IgA-peptide (A, B) and Tx45-IgM-peptide (C, D) derived cholera proteins and sera of mice immunized with oral vaccine (15 days after vaccination (A, C) and 30 days after vaccination (B, D).

Figure S3: List of single (TxA2-A, TxB3-A, Tx-P-6A), multi antigen peptides (MAP4) (TxA-2M, TxA-3M, TxA-6M) and tripeptides synthetic peptides (Tx45-IgA, Tx45-IgM) and physicochemical properties.
