# Supplementary material for: Dynamics of IgM and IgA Antibody Response Profile Against Vibrio cholerae Toxins A, B, and P
Source: Int J Mol Sci. 2025 Apr 9;26(8):3507. doi: 10.3390/ijms26083507 (PMC12027190; doi:10.3390/ijms26083507)
Supplement: Supplementary file 1 [file ijms-26-03507-s001.zip › ijms-3387352-supplementary.pdf]

Spot	Sequence	Spot	Sequence	Spot	Sequence
A1	GSGSGMVKIIFVFFI	B1	NDVLGAYSPHPDEQE	C1	VKRQIFSGYQSDIDT
A2	MVKIIFVFFIFLSSF	B2	AYSPHPDEQEVSALG	C2	FSGYQSDIDTHNRIK
A3	FVFFIFLSSFSYAND	B3	PDEQEVSALGGIPYS	C3	SDIDTHNRIKDELGS
A4	FLSSFSYANDDKLYR	B4	VSALGGIPYSQIYGW	C4	DTHNRIKDELGSGSG
A5	SYANDDKLYRADSRP	B5	GIPYSQIYGWYRVHF	C5	GSGSGMIKLKFGVFF
A6	DKLYRADSRPPDEIK	B6	QIYGWYRVHFGVLDE	C6	MIKLKFGVFFTVLLS
A7	ADSRPPDEIKQSGGL	B7	YRVHFGVLDEQLHRN	C7	FGVFFTVLLSSAYAH
A8	PDEIKQSGGLMPRGQ	B8	GVLDEQLHRNRGYRD	C8	TVLLSSAYAHGTPQN
A9	QSGGLMPRGQSEYFD	B9	QLHRNRGYRDRYYSN	C9	SAYAHGTPQNITDLC
A10	MPRGQSEYFDRGTQM	B10	RGYRDRYYSNLDIAP	C10	GTPQNITDLCAEYHN
A11	SEYFDRGTQMNINLY	B11	RYYSNLDIAPAADGY	C11	ITDLCAEYHNTQIYT
A12	RGTQMNINLYDHARG	B12	LDIAPAADGYGLAGF	C12	AEYHNTQIYTLNDKI
A13	NINLYDHARGTQTGF	B13	AADGYGLAGFPPEHR	C13	TQIYTLNDKIFSYTE
A14	DHARGTQTGFVRHDD	B14	GLAGFPPEHRAWREE	C14	LNDKIFSYTESLAGK
A15	TQTGFVRHDDGYVST	B15	PPEHRAWREEPWIHH	C15	FSYTESLAGKREMAI
A16	VRHDDGYVSTSISLR	B16	AWREEPWIHHAPPGC	C16	SLAGKREMAIITFKN
A17	GYVSTSISLRSAHLV	B17	PWIHHAPPGCGNAPR	C17	REMAIITFKNGAIFQ
A18	SISLRSAHLVGQTIL	B18	APPGCGNAPRSSMSN	C18	ITFKNGAIFQVEVPG
A19	SAHLVGQTILSGHST	B19	GNAPRSSMSNTCDEK	C19	GAIFQVEVPGSQHID
A20	GQTILSGHSTYYIYV	B20	SSMSNTCDEKTQSLG	C20	VEVPGSQHIDSQKKA
A21	SGHSTYYIYVIATAP	B21	TCDEKTQSLGVKFLD	C21	SQHIDSQKKAIERMK
A22	YYIYVIATAPNMFNV	B22	TQSLGVKFLDEYQSK	C22	SQKKAIERMKDTLRI
A23	IATAPNMFNVNDVLG	B23	VKFLDEYQSKVKRQI	C23	IERMKDTLRIAYLTE
A24	NMFNVNDVLGAYSPH	B24	EYQSKVKRQIFSGYQ	C24	DTLRIAYLTEAKVEK
D1	AYLTEAKVEKLCVWN	E1	DEHKTLIENVKLQGY	F1	YEQKTLECTKNGSGS
D2	AKVEKLCVWNNKTPH	E2	LIENVKLQGYRINII	F2	EQKTLECTKNGSGSG
D3	LCVWNNKTPHAIAAI	E3	KLQGYRINIIQVIVS	F3	
D4	NKTPHAIAAISMANG	E4	RINIIQVIVSENVVD	F4	
D5	HAIAAISMANGSGSG	E5	QVIVSENVVDEADCS	F5	
D6	GSGSGMGYVRVIYQF	E6	ENVVDEADCSQKKSV	F6	
D7	MGYVRVIYQFPDNLW	E7	EADCSQKKSVKERIK	F7	
D8	VIYQFPDNLWWNECT	E8	QKKSVKERIKIEWGK	F8	
D9	PDNLWWNECTNQVYY	E9	KERIKIEWGKINVVP	F9	
D10	WNECTNQVYYAQDPM	E10	IEWGKINVVPYLVFS	F10	
D11	NQVYYAQDPMKPERL	E11	INVVPYLVFSALYVA	F11	
D12	AQDPMKPERLIGTPS	E12	YLVFSALYVALLPVI	F12	
D13	KPERLIGTPSIIQTK	E13	ALYVALLPVIWWSYG	F13	
D14	IGTPSIIQTKLLKIL	E14	LLPVIWWSYGQWYQH	F14	
D15	IIQTKLLKILCEYHP	E15	WWSYGQWYQHELAGI	F15	
D16	LLKILCEYHPAPCPN	E16	QWYQHELAGITHDLR	F16	
D17	CEYHPAPCPNDQIIK	E17	ELAGITHDLRDLARL	F17	
D18	APCPNDQIIKALWPH	E18	THDLRDLARLPGITI	F18	
D19	DQIIKALWPHGFISS	E19	DLARLPGITIQKLSE	F19	
D20	ALWPHGFISSESLTQ	E20	PGITIQKLSEQKLTF	F20	
D21	GFISSESLTQAIKRT	E21	QKLSEQKLTFAIDQH	F21	
D22	ESLTQAIKRTRDFLN	E22	QKLTFAIDQHQCSVN	F22	
D23	AIKRTRDFLNDEHKT	E23	FVKNPTDTGHGTVV	F23	
D24	RDFLNDEHKTLIENV	E24	TDTGHGTVVMQVKV	F24	
G1		G9	QEVRKYFCV	G17	QEVRKYFCV
G2		G10		G18	
G3	KEVPALTAVETGATN	G11	KEVPALTAVETGATN	G19	
G4		G12		G20	
G5	GYPKDGNAFNNLDRI	G13	GYPKDGNAFNNLDRI	G21	
G6		G14		G22	
G7	YDYDVPDYAGYPYDV	G15	YDYDVPDYAGYPYDV	G23	
G8		G16		G24	YDYDVPDYAGYPYDV

Code	Sequence	Type	MM	Charge (pH 7)	pI (pH7)
TxA-2A	YRVHFGVLDEQLHRN	MAP4	7934.91	0.2	7.68
TxB-3A	*GG*REMAIITFKNGGG	MAP4	6433.45	+1	10.11
TxP-6A	GGAIKRTRDELNGGG	MAP4	6405,05	+1	10.1
TxA-2M	GGMPRGQSEYFDGGG	MAP4	6460.89	-1	3.93
TxA-3M	GGGNINLYDHARGGG	MAP4	6232.61	0.1	7.75
TxA-6M	NMFNVNDVLGAYSPH	MAP4	6715.42	-0.9	4.87
Tx-45A	YRVHFGVLDEQLHRNGGREMAIITEKNGGGGGAIKRTRDFLNGGG	45 mer	4855.44	2.2	10.16
Tx-45M	GGMPRGQSEYFDGGGGGGNINLYDHARGGGNMFNVNDVLGAISPH	45 mer	4563.93	-1.8	5.8

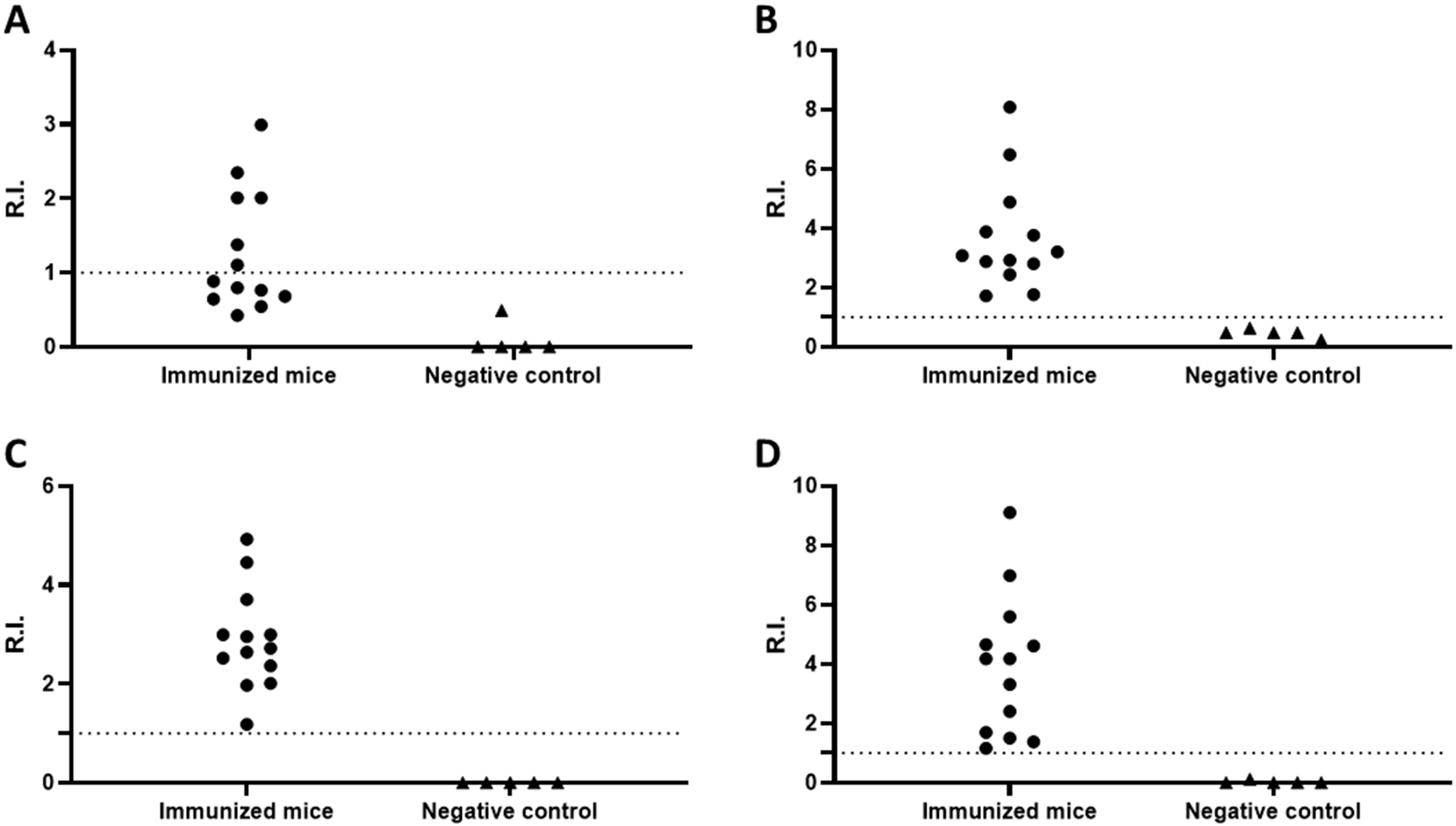
A
B
C
D
R.I.
Immunized mice
Negative control
0
1
2
3
4
6
8
10
